# Energetics and Electronic Structure of h-BN Nanoflakes

**DOI:** 10.1038/srep30653

**Published:** 2016-08-02

**Authors:** Ayaka Yamanaka, Susumu Okada

**Affiliations:** 1 Graduate School of Pure and Applied Sciences, University of Tsukuba, 1-1-1 Tennodai, Tsukuba, Ibaraki 305-8571, Japan

## Abstract

We studied the energetics and electronic structure of hexagonal boron nitride (h-BN) nanoribbons with hydrogenated and clean edges with respect to the detailed edge shapes using density functional theory. Our calculations showed that the stability of h-BN edges strongly depends on the edge termination. In the case of hydrogenated edges, the formation energy is constant for all edge angles ranging from armchair to zigzag, indicating that h-BN may exhibit rich variation in their edge atomic arrangements under static conditions. The hydrogenated h-BN nanoribbons are insulators with an energy gap of 4 eV irrespective of edge shape, in which the lowest branch of the conduction band exhibits nearly free electron states nature distributed in the vacuum region outside the ribbons. In contrast, the formation energy of h-BN nanoribbons with clean edges monotonically increases as the edge angle is changed from armchair to zigzag. Our analysis reveals that the increase of density of states at the Fermi level arising from dangling bond states leads to this monotonic increase of edge formation energy in h-BN nanoribbons with clean edges.

Hexagonal boron nitride (h-BN) is known to be a prototypical layered material in which each layer is composed of B and N atoms arranged in a hexagonal network like the C atoms of graphite^1^,^2^,^3^. Along the direction normal to each layer, in sharp contrast to graphite, each layer is weakly bound in an AA′ arrangement, in which N atoms are situated just above/below B atoms in adjacent layers, and *vice versa*, due to the interlayer Coulomb interaction between B and N atoms^4^. The chemical difference between B and N atoms makes h-BN an insulator with a large energy gap of 5 eV between the valence-band top (VBT) and conduction-band bottom (CBB) at a K point localized on N and B atoms, respectively^5^,^6^. Due to its atomically flat network, h-BN has been used as the supporting materials for graphene devices that exhibit remarkable carrier mobility^7^,^8^,^9^,^10^. On the other hand, h-BN itself is attracting attention because of its structural similarity to graphene^11^,^12^,^13^,^14^. By the analogy with the graphene, h-BN can form various derivatives applicable to wide-ranging areas of modern technology: Nanoscale tubes and flakes of h-BN have been synthesized by rolling or cutting h-BN sheets with appropriate boundary conditions^15^,^16^,^17^,^18^,^19^,^20^,^21^,^22^,^23^,^24^,^25^. For many of the applications of nanoscale h-BN derivatives, it is critical to precisely control their geometric and electronic structures. In particular, the energetics of the nanoflakes yields fundamental insights into the practical procedures to control the structures of nanoscale h-BN derivatives.

Ribbons with nanometer width are a relevant structural model to investigate the energetics and electronic structures of h-BN nanoflakes. Several studies have demonstrated the stability and electronic structures of h-BN nanoribbons with armchair and zigzag edges^26^,^27^,^28^,^29^,^30^,^31^. However, the stability and electronic structure of h-BN with chiral edges are still unknown because these h-BN nanoflakes may possess edges with arbitrary shapes. In the case of graphene nanoribbons, energetics and electronic structure strongly correlate with their detailed edge structure and edge termination^32^. Because of the structural similarity between h-BN and graphene, an analogous correlation between the edge geometries and physical properties of h-BN nanoribbons with arbitrary edge shapes to that of graphene is expected.

In this paper, we aim to elucidate the correlation between the edge shape and physical properties of hydrogenated and clean h-BN nanoribbons with various edge shapes, including armchair, zigzag, and chiral edges, using first-principles total-energy calculations based on density functional theory. Our calculations show that the energetics and electronic structure of h-BN nanoribbons strongly depend on the edge termination. In the case of hydrogenated edges, we found that the formation energy is constant for all edge angles between armchair and zigzag. We also found that all ribbons are insulators with an energy gap of 4 eV. The VBT is primarily distributed on N atoms extended throughout the ribbon. The CBB exhibits nearly free electron (NFE) state nature, which is distributed in the vacuum region alongside the N-rich edges of the nanoribbons, except for those with near-armchair edges. For ribbons with near-armchair edges, the CBB is distributed in the vacuum region above and below the atomic layer. In contrast, the formation energy of h-BN nanoribbons with clean edges monotonically increases as the edge angle changes from armchair to zigzag. Our analysis reveals that the increase of the density of states (DOS) at the Fermi level *E*_*F*_ arising from dangling bond states leads to the monotonic increase of edge formation energy of the h-BN nanoribbons with clean edges.

## Results

**Figure 1:**
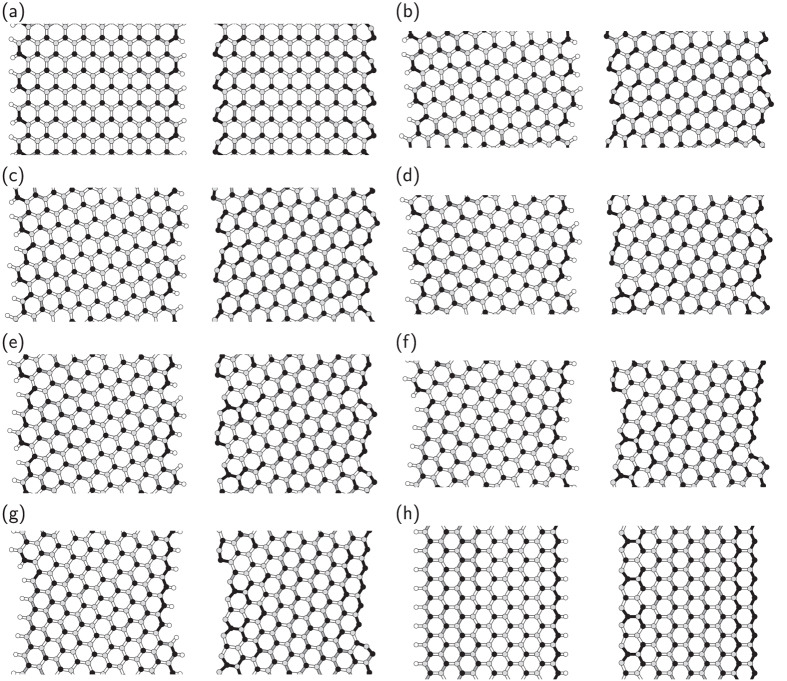
Geometric structure of h-BN nanoribbons. Optimized geometries of h-BN nanoribbons with (**a**) an armchair edge (*θ* = 0°), chiral edges with (**b**) *θ* = 5°, (**c**) *θ* = 8°, (**d**) *θ* = 14°, (**e**) *θ* = 16°, (**f**) *θ* = 22°, (**g**) *θ* = 23°, and (**h**) a zigzag edge (*θ* = 30°). In each figure, left and right panels denote the h-BN nanoribbons with hydrogenated and clean edges, respectively. Black, gray, and white circles denote nitrogen, boron, and hydrogen atoms, respectively. Black, gray, and white bonds indicate short (−1.44 Å), medium (1.44–1.45 Å), and long (1.45 Å–) bonds, respectively. White bonds situated at the edge of the nanoribbons correspond to B–H and N–H bonds.

We considered several edge structures between armchair and zigzag of h-BN to investigate their energetics and electronic structures. To simulate h-BN edges with various edge shapes, we considered nanoribbons with hydrogenated and clean edges with edge angles of 0 (armchair), 5, 8, 14, 16, 22, 23, and 30° (zigzag) (Fig. 1). To allow quantitative discussion of the energetics of the nanoribbons with various edge shapes, the ribbons possessed similar widths and unit lengths of about 1.8–2.2 and 1.5–2.0 nm, respectively. The geometric structures of h-BN nanoribbons were optimized until the force acting on atoms was less than 0.005 Ry/Å under the fixed lattice parameter along the ribbons, which was determined by the bulk bond length of 1.45 Å.

### Hydrogenated edges

Figure 1 shows the optimized structures of h-BN nanoribbons with various edge angles. In all cases, the bond lengths of the nanoribbons are not equivalent. Bonds associated with hydrogenated N atoms are shorter than the other bonds. The bond lengths around hydrogenated N atoms are 1.44 Å or shorter due to the inward structural reconstruction increasing the *π* nature of edge N atoms to accommodate excess electrons provided by H atoms. For a ribbon with armchair edges, there is a symmetric bond alternation along the ribbon direction. In contrast, for other ribbons, bond alternation is asymmetric because of the asymmetric atomic arrangement at edges. In particular, significant asymmetric structural reconstruction occurs in the ribbons with zigzag edges. In this case, the bond length retains its bulk value near the N edge while substantial bond alternation occurs near the B edge.

**Figure 2:**
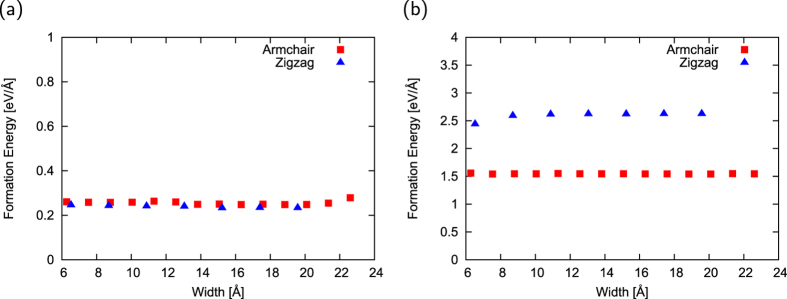
Width dependence of edge formation energy of h-BN nanoribbons. Edge formation energy of h-BN nanoribbons with (**a**) hydrogenated and (**b**) clean edges as a function of ribbon width. Red squares and blue triangles denote the edge formation energy of nanoribbons with armchair and zigzag edges, respectively.

Before investigating the energetics of h-BN ribbons with hydrogenated edges with arbitrary edge shapes, we investigated the edge formation energy of h-BN nanoribbons with armchair and zigzag edges with respect to ribbon width, because the edge formation energy of graphene nanoribbons strongly depends on their width^33^. Figure 2(a) shows the edge formation energy of hydrogenated h-BN nanoribbons as a function of ribbon width. The edge formation energy *E*_edge_ was evaluated using the following formula:


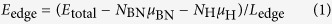


where *E*_total_, *N*_BN_, *N*_H_, *μ*_BN_, *μ*_H_, and *L*_edge_ denote the total energy of ribbons, the number of pairs of B and N atoms, the number of H atoms, the energy potential of h-BN per BN pair, the chemical potential of H atoms, and the edge length of a unit cell, respectively. *μ*_H_ is evaluated by the total energy per atom of H_2_ molecules. As shown in Fig. 2(a), edge formation energy of the hydrogenated h-BN nanoribbons is almost constant up to a width of 22 Å for both zigzag and armchair ribbons. Thus, the edge formation energy of the H-terminated h-BN nanoribbons is nearly independent of the ribbon width and edge shape. Note that the edge formation energy contains a numerical error of 5 meV, which arises from the total energies of ribbons with different numbers of BN pairs depending on their unit cell size.

**Figure 3:**
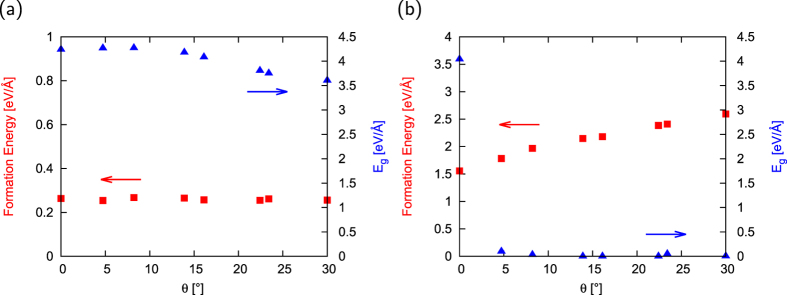
Edge angle dependence of edge formation energy of h-BN nanoribbons. Edge formation energy and energy gap of h-BN nanoribbons with (**a**) hydrogenated and (**b**) clean edges as a function of edge angle *θ*.

Although the edge energy of the hydrogenated h-BN nanoribbon with zigzag edges is almost the same as that with armchair edges, it is worth investigating the detailed edge angle dependence of the edge formation energy. Figure 3(a) shows the edge formation energy and the energy gap between VBT and CBB of hydrogenated h-BN nanoribbons as a function of the edge angle. As shown in Fig. 3(a), the edge formation energy retains a constant value for all edge angles. Thus, based on the energetics, *i.e*. the constant edge formation energy, h-BN flakes are unlikely to possess preferential edge shape under static conditions. This result implies that the h-BN flakes inherently possess edge roughness. The h-BN nanoribbons are insulators with an energy gap of about 4 eV, irrespective of the edge angle *θ*. In the case of graphene nanoribbons with hydrogenated edges, the edge formation energy retains constant up to the angle *θ* = 16°, where the ribbons possess semiconducting electronic structure with a finite energy gap. Thus, the constant edge formation energy of the h-BN nanoribbons is ascribed to their insulating electronic structure.

**Figure 4:**
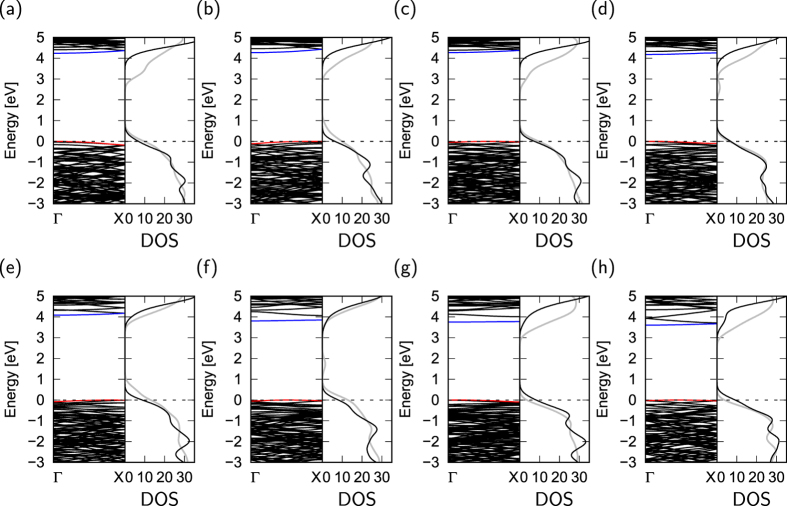
Electronic structure of hydrogenated h-BN nanoribbons. Electronic structure and density of states (DOS) of hydrogenated h-BN nanoribbons with edge angles *θ* of (**a**) 0°, (**b**) 5°, (**c**) 8°, (**d**) 14°, (**e**) 16°, (**f**) 22°, (**g**) 23°, and (**h**) 30°. Red and blue lines indicate the VBT and CBB states, respectively. Gray lines denote the DOS at the temperature of 1000 K. Energies are measured from the VBT.

Figure 4 shows the electronic energy bands and DOS of hydrogenated h-BN nanoribbons with various edge angles. All ribbons are insulators with an energy gap of about 4 eV. This energy gap is considerably narrower than that of bulk h-BN, indicating that the detailed electronic properties of h-BN ribbons around the gap are different from those of bulk h-BN. As shown in Fig. 4, the CBB retains a constant value up to the angle of 8°, and then gradually shifts downward with increasing the edge angle *θ*, resulting in a decrease of the energy gap. The dependence of the electronic structure on edge angle implies that the polarization of h-BN around its edges plays an important role in determining the positions of the lower branches of the conduction band. For the ribbons with *θ* = 14° or larger, the lowest branch of the conduction band is separated from the bulk states, leading to an additional structure in the DOS of unoccupied states. It is worth investigating how the electronic structures of h-BN nanoribbons depend on the structural corrugation or rippling formed at elevated temperature. To check the DOS of the ribbons at elevated temperature, we performed *ab initio* molecular dynamics calculations at 1000 K. After a simulation time of 100 fs, all h-BN nanoribbons possess structural corrugation of up to 2 Å. The structural corrugation due to the finite temperature decreases the band gap of the nanoribbons, but does not affect the qualitative shape of the DOS.

**Figure 5:**
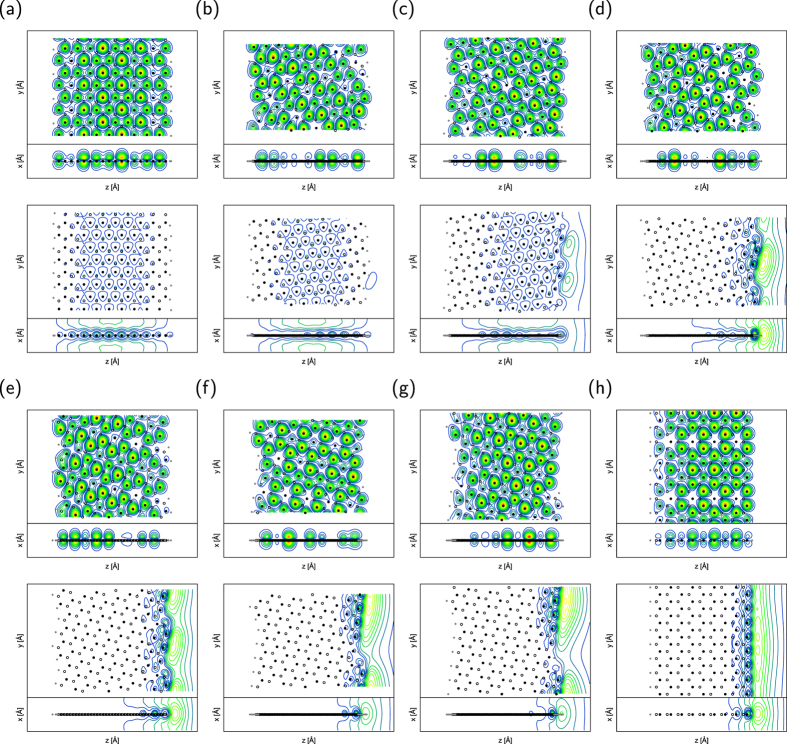
Wave functions of hydrogenated h-BN nanoribbons. Top (*x* = 0) and side (*y* = 0) views of contour plots of wave functions at the Γ point near the Fermi level *E*_*F*_ of hydrogenated h-BN nanoribbons with edge angles *θ* of (**a**) 0°, (**b**) 5°, (**c**) 8°, (**d**) 14°, (**e**) 16°, (**f**) 22°, (**g**) 23°, and (**h**) 30°. In each figure, upper and lower panels denote VBT and CBB states, respectively. Black, white, and gray circles denote the atomic positions of nitrogen, boron, and hydrogen atoms, respectively.

To unravel the physical origin of the detailed electronic structure modulation in the lower branches of the conduction band with respect to the edge angle, we investigated the wave function distribution of VBT and CBB states at the Γ point. Figure 5 presents the contour plots of squared wave functions of the VBT and CBB states of h-BN with various edge angles at the Γ point. For the h-BN ribbons with all edge angles, the VBT state is distributed on N atoms and extended throughout the ribbon. The distribution is qualitatively the same as that of the VBT of bulk h-BN. On the other hand, the distribution exhibits different characteristics to that of graphene nanoribbons. In the case of graphene nanoribbons, except for those with armchair edges, the VBT state is localized at the edge atomic sites with zigzag structure because of its edge state nature^32^. However, as shown in Fig. 5, the VBT state of the h-BN nanoribbon does not exhibit an edge-localized nature even though the ribbon has perfect zigzag edges.

In contrast to the VBT state, the CBB state exhibits an unusual nature, which is totally different from those of bulk h-BN and graphene nanoribbons. The CBB state of all h-BN ribbons is not distributed on the atomic sites but in the vacuum region where atoms are absent, exhibiting the NFE state nature which is inherent in the layered materials, such as graphene^34^,^35^,^36^,^37^,^38^, h-BN^5^,^6^, and transition metal dichalcogenides^39^. For the ribbons with edge angles up to 8°, CBB states are distributed in the vacuum region above and below the ribbons, similar to the conventional NFE states of h-BN sheets and graphene. For ribbons with zigzag and near-zigzag edges, the maxima of the CBB states are distributed alongside the rightmost edges of the ribbons with about 3 Å vacuum region. The states are primarily distributed in the vacuum region separated from the rightmost edge atomic site and are also slightly distributed at the atomic site near the edge. Furthermore, the states are extended along the direction parallel to the ribbon with small undulations in the vacuum region that reflect the edge atomic arrangement. The characteristic distribution of these states as well as the quadratic dispersion band indicate that the CBB states of ribbons with near-zigzag and zigzag edges are the NFE states at the edge of the atomic networks, as in the case of graphene nanoribbons under a lateral electric field^37^,^38^. Calculating the effective electron mass from the energy band revealed that the effective masses of nanoribbons are ranging from 0.9 to 1.1 *m*_*e*_, where *m*_*e*_ is the bare electron mass. Thus, the lowest branch of the conduction band of h-BN nanoribbons still possesses NFE nature. Furthermore, because the NFE nature is sensitive to the electrostatic potential, the CBB state with edge NFE nature shifts downward with increasing edge angle, corresponding to an increase in the number of N atoms that deepen the electrostatic potential outside the ribbon.

### Clean edges

As shown in Fig. 1, substantial structural reconstruction occurs at edge atoms to reduce electron energy arising from the dangling bonds with increasing lattice energies in all nanoribbons except the edge angle of 30° (zigzag). In particular, atoms situated at the armchair portion are remarkably deformed from the ideal sp^2^ bond angles by the reconstruction. N atoms protrude from the edges to increase their 2*s* nature to accommodate excess electrons. According to the protruding nature of N atoms, B atoms shift inward to form a linear sp-hybridized chain with neighboring N atoms supplying their valence electrons to N atoms. In addition to the substantial structural reconstruction, the ribbon with near-zigzag edges exhibits bond alternation both along and normal to the ribbon. For the ribbon with zigzag edges, the bond alternation normal to the ribbon occurs around the N edge. On the other hand, around the B edge, long-range bond modulation occurs along the ribbon direction.

Because of the dangling/unsaturated bonds at the edge atomic sites, it is thought that the clean edges are less stable than the hydrogenated edges. Figure 2(b) shows the edge formation energy of h-BN nanoribbons with clean edges as a function of ribbon width. The edge formation energy *E*_edge_ was evaluated using the following formula:


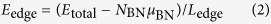


where the terms have the same meanings as above. As shown in Fig. 2(b), the formation energy of clean edges is six to ten times larger than that of hydrogenated edges. In contrast to the nanoribbons with hydrogenated edges, edge formation energy of armchair edges is smaller than that of zigzag edges for the h-BN nanoribbons with clean edges. This indicates that in the case of h-BN nanoribbons with clean edges, the armchair edges are more stable than the zigzag edges because the large structural reconstruction decreases the electron energy arising from the unsaturated B/N bonds. On the other hand, the edge formation energy remains constant in the ribbons with a width of 7 Å or wider as in the case of hydrogenated edges.

Figure 3(b) shows the edge formation energy and the energy gap of h-BN nanoribbons with clean edges as a function of the edge angle. In contrast to nanoribbons with hydrogenated edges, the edge formation energy monotonically increases with increasing the edge angle without any plateaus. By analogy with the relationship between edge formation energies and electronic structure in graphene nanoribbons^32^, the monotonic increase of the edge formation energy implies that nanoribbons with clean edges are metals with a number of electron states at *E*_*F*_.

We also found that the ribbons with armchair edges and an edge angle of *θ* = 5° are semiconductors while the other ribbons are metals, in contrast to nanoribbons with hydrogenated edges. The ribbons with an edge angle of 0 and 5° have fundamental gaps of 4 and 0.1 eV, respectively. The semiconducting nature of the armchair ribbons makes the armchair edge the most energetically stable among the eight edge angles. The semiconducting electronic structure of the ribbon with armchair edges is ascribed to the substantial atomic reconstruction at its edges. Because of this reconstruction, B atoms at the armchair edge possess an sp nature with empty *π* states, while N atoms possess decomposed s and p states that are fully filled by electrons. In accordance with the reconstruction, this nanoribbon does not possess unsaturated bonds even though it is not terminated by H atoms. By focusing on the detailed edge atomic arrangement of the ribbons with respect to the edge angle, we find that substantial structural reconstruction occurs around the armchair regions of the edges. Thus, the monotonic increase in the edge formation energy is ascribed to the decrease of armchair portions. As shown if Fig. 3(b), as in the case of hydrogenated edges, there is a correlation between edge formation energy and energy gap. Semiconducting armchair nanoribbons have small edge formation energies while metallic nanoribbons have edge formation energies larger than that of the armchair nanoribbon.

**Figure 6:**
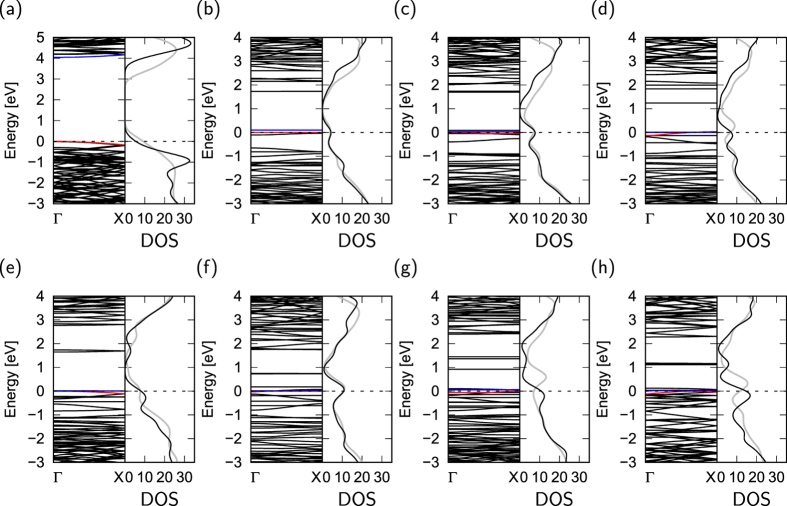
Electronic structure of h-BN nanoribbons with clean edges. Electronic structures and density of states (DOS) of h-BN nanoribbons with clean edges of which edge angles are (**a**) 0°, (**b**) 5°, (**c**) 8°, (**d**) 14°, (**e**) 16°, (**f**) 22°, (**g**) 23°, and (**h**) 30°. Red and blue lines indicate VBT and CBB states, respectively. Gray lines denote the DOS of the nanoribbons at the temperature of 1000 K. Energies are measured from *E*_*F*_ and VBT for metallic and semiconducting nanoribbons, respectively.

Figure 6 shows the electronic energy band structure and DOS of nanoribbons with clean edges. Compared with the electronic structures of nanoribbons with hydrogenated edges, nanoribbons with clean edges have extra states around *E*_*F*_ with less dispersion, arising from the dangling/unsaturated bonds of B and N atoms situated at the edges. Because of the localized nature of the dangling bond states, these states cause the flat dispersion band at *E*_*F*_. Because of the substantial atomic reconstruction, the dangling bonds are absent at the atomic sites at the armchair edges. Thus, the number of states at *E*_*F*_ increases with increasing proportion of zigzag edges. Indeed, the DOS at *E*_*F*_ monotonically increases with increasing edge angle *θ* from 5 to 30°. This large number of states at *E*_*F*_ causes instability in the energy of the edges with these angles, similar to the case of graphene nanoribbons. As in the case of nanoribbons with hydrogenated edges, the electronic structure of nanoribbons with clean edges is less sensitive to the structural corrugations under the finite temperature.

**Figure 7:**
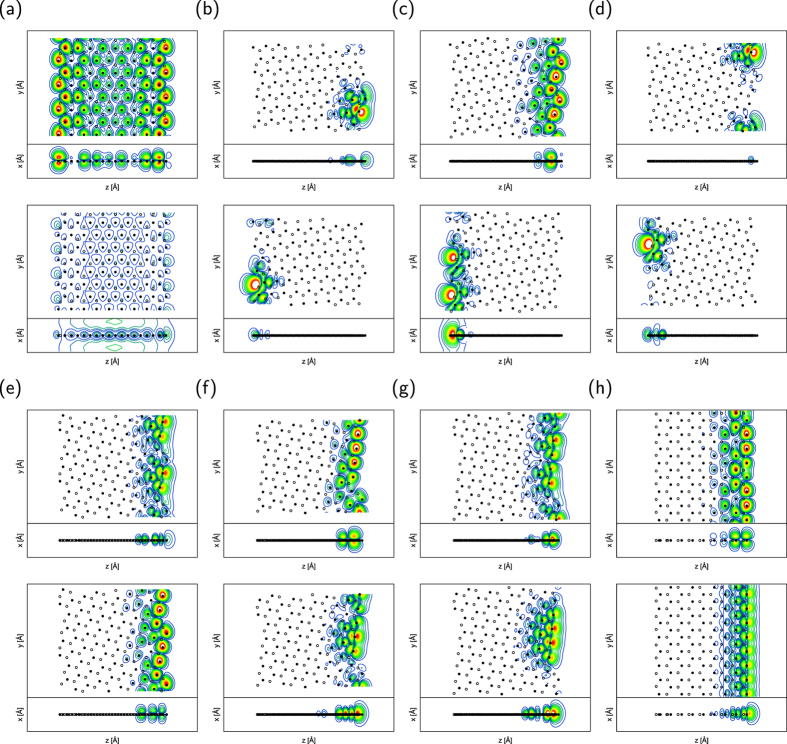
Wave functions of h-BN nanoribbons with clean edges. Top (*x* = 0) and side (*y* = 0) views of contour plots of wave functions at the Γ point near the Fermi level *E*_*F*_ of h-BN nanoribbons with clean edges of which edge angles *θ* of (**a**) 0°, (**b**) 5°, (**c**) 8°, (**d**) 14°, (**e**) 16°, (**f**) 22°, (**g**) 23°, and (**h**) 30°. In each figure, upper and lower panels denote VBT and CBB states, respectively. Black and white circles denote the atomic positions of nitrogen and boron atoms, respectively.

It is worth investigating the detailed properties of the electronic states at or near *E*_*F*_. To unravel the origin of these states, we depict the squared wave function of the electron states of nanoribbons with edge angles of 0, 5, 8, 14, 16, 22, 23, and 30° at or near *E*_*F*_ in Fig. 7 at the Γ point. For the nanoribbon with armchair edges, its VBT state is distributed on N atoms with *π* nature and extends throughout the ribbons. The CBB state has an NFE nature, which is distributed in the vacuum region above and below the ribbon, as in the case of the nanoribbons with hydrogenated edges. For the remaining ribbons, the state at *E*_*F*_ is primarily localized on N atoms situated near the N-rich edges with *π* and *σ* natures. By focusing on the wave function of the ribbon with zigzag edges, we find that VBT and CBB states are localized at the N atoms at the N-rich edge with *σ* and *π* natures, respectively. Thus, the states with *σ* nature are classified as dangling bond states arising from the unsaturated bond of N atoms at the edges with zigzag shapes. It should be noted that such states are absent at the atomic sites of edges with armchair shapes. In this case, the edge reconstruction at the armchair region leads to substantial upward and downward shifts of states induced by a considerable change of orbital hybridization. In contrast to the ribbons with hydrogenated edges, no delocalized states with NFE nature emerge at or near *E*_*F*_ for the ribbons with finite edge angles.

## Discussion

We studied the geometric and electronic structures of h-BN nanoribbons with edge angles ranging from armchair to zigzag using density functional theory. Our calculations show that the edge stability and electronic structure of h-BN nanoribbons strongly depend on the edge termination. In the case of hydrogenated edges, the edge formation energy retains a constant value for all nanoribbons. This indicates that hydrogenated h-BN nanoribbons and nanoflakes inherently possess edge roughnesses under static conditions. On the other hand, for ribbons with clean edges, the edge formation energy monotonically increases with the proportion of zigzag edge. Furthermore, the edge stability strongly correlates with the electronic structure of h-BN nanoribbons. Nanoribbons with small edge formation energy are semiconductors with a finite energy gap, while ribbons with large formation energy are metals with a large DOS at *E*_*F*_. By analyzing the wave functions near *E*_*F*_, we found that the dangling bond states appeared around *E*_*F*_ for nanoribbons with clean edges, except for that with armchair edges. Based on these findings, the increase in the DOS near *E*_*F*_ arising from the dangling bond states is the origin of the increase in formation energy for clean edges. The present results indicate that the shape of the h-BN nanoflakes is tunable by controlling the edge termination by atoms and molecules. Furthermore, the energetics provides guiding principles to design heterogeneous layered materials consisting of h-BN and graphene that possess unusual electronic structures^40^,^41^.

## Methods

The geometric and electronic structures of h-BN nanoribbons with various edge shapes were studied using density functional theory^42^,^43^ implemented into the Simulation Tool for Atom Technology (STATE)^44^. We used the generalized gradient approximation with the Perdew-Burke-Ernzerhof functional form^45^,^46^. Ultrasoft pseudopotentials generated by the Vanderbilt scheme were used to describe electron-ion interactions^47^. Valence wave functions and charge densities were expanded in terms of the plane wave basis set with cutoff energies of 25 and 225 Ry, respectively. Brillouin zone integration was carried out using equidistance 1 *k*-point along the ribbon direction, which corresponds to the eight *k*-points sampling in the conventional cell of h-BN, resulting in sufficient convergence in the total energy of the electronic structure of the ribbons.

The effective screening medium (ESM) method was adopted to avoid unphysical dipole interactions with the periodic images arising from their polar edges in the framework of the conventional DFT calculations^48^. This is because h-BN nanoribbons with arbitrary edge shapes intrinsically possess lateral polarization arising from the chemical difference between B and N atoms. In this case, to simulate the open boundary condition in lateral inter-ribbon directions, we put ESM with a relative permittivity of 1, which simulates vacuum conditions in this region (*ε*_0_ = 8.854 × 10^−12^ Fm^−1^), at the cell boundaries with vacuum spacing of 8 Å to the rightmost and leftmost atoms of the nanoribbons.

## Additional Information

**How to cite this article**: Yamanaka, A. and Okada, S. Energetics and Electronic Structure of h-BN Nanoflakes. *Sci. Rep*. **6**, 30653; doi: 10.1038/srep30653 (2016).
